# The Role and Interactions of Programmed Cell Death 4 and its Regulation by microRNA in Transformed Cells of the Gastrointestinal Tract

**DOI:** 10.3389/fonc.2022.903374

**Published:** 2022-06-29

**Authors:** William Frank Ferris

**Affiliations:** Division of Clinical Pharmacology, Department of Medicine, Faculty of Medicine and Health Sciences, Stellenbosch University, Tygerberg, South Africa

**Keywords:** PDCD4, microRNA, gastrointestinal tract, oesophageal cancer, gastric cancer, colorectal cancer

## Abstract

Data from GLOBOCAN 2020 estimates that there were 19.3 million new cases of cancer and 10.0 million cancer-related deaths in 2020 and that this is predicted to increase by 47% in 2040. The combined burden of cancers of the gastrointestinal (GI) tract, including oesophageal-, gastric- and colorectal cancers, resulted in 22.6% of the cancer-related deaths in 2020 and 18.7% of new diagnosed cases. Understanding the aetiology of GI tract cancers should have a major impact on future therapies and lessen this substantial burden of disease. Many cancers of the GI tract have suppression of the tumour suppressor Programmed Cell Death 4 (PDCD4) and this has been linked to the expression of microRNAs which bind to the untranslated region of PDCD4 mRNA and either inhibit translation or target the mRNA for degradation. This review highlights the properties of PDCD4 and documents the evidence for the regulation of PDCD4 expression by microRNAs in cancers of the GI tract.

## Introduction

Cancers of the gastrointestinal (GI) tract constitute a substantial portion of the global burden of disease with oesophageal-, gastric- and colorectal cancers being the 7^th^ (at 3.1%), fifth (at 5.6%) and third (at 10.0%) the most prevalent in 2020 respectively. The often-late presentation, mainly due to a lack of symptoms in early-stage disease, results in a high mortality rate with oesophageal -, gastric- and colorectal cancers having the sixth (at 5.5%), fourth (at 7.7%) and second (at 9.4%) most cancer deaths (1). Analysis of many GI cancers has revealed downregulation of the tumour suppressor Programmed Cell Death 4 (PDCD4), which is often associated with accelerated disease progression and a poor prognosis. Although changes in the expression and intracellular location of the protein have extensively been linked with cellular dysregulation, no explicit function of PDCD4 has been directly connected with the maintenance of quiescence. However, the burgeoning number of publications examining changes in the expression of PDCD4 in transformed cells and tumours exemplifies the potential importance of this protein in cell regulation. The downregulation of the tumour repressor may therefore be indicative of the state of transformation in many cells and ultimately patient prognosis. Understanding the regulation of PDCD4 expression should increase our insight into the aetiology and progression of many GI cancers.

## PDCD4 as a Tumour Suppressor

Programmed Cell Death 4 (PDCD4 - previously also known as H731, TIS, 195/15a, MA-3) is ubiquitously expressed and was so named as the protein was initially found to be associated with apoptosis (2, 3). The gene is highly conserved and is found in human, mouse, chicken, Xenopus, Drosophila and the marine sponge *S. domuncula* (3), implying that the protein performs an essential function.

There are a multitude of studies examining the expression of PDCD4 in cancer or carcinoid cells. The protein is frequently down-regulated in renal, lung and glia-derived cancers (4, 5), squamous cell carcinomas of the mouth (6), poorly differentiated nasopharyngeal cancers (7), lymphomas (8), cancers of the breast (9) and ovary (10, 11), oesophageal cancer (12, 13), gastric cancer (14, 15) and in colon carcinoma (16, 17). Moreover, meta-analysis has confirmed that low PDCD4 expression is strongly associated with the differentiation status of solid tumours and with tumour size (18). However, PDCD4 is not always downregulated in cancers (5), as expression has been found to be increased, decreased or remain unchanged in squamous lung carcinoma (19) and it is highly expressed in proliferating cells in some carcinomas of the bladder and breast (20). In addition, a study by Goa et al. showed that only 47% of the 30 gliomas tested had reduced PDCD4 mRNA, whereas 77% had a loss of protein, with no correlation between expression and either pathological or clinical features of the tumours (21). The lack of correlation between PDCD4 mRNA or protein expression has also been noted in some lung carcinomas (19). Although not all cancers exhibit decreased PDCD4 expression, which diminishes the potential use of PDCD4 expression as a diagnostic tool, decreased expression is very frequently associated with poor prognosis.

Evidence of PDCD4 as a tumour suppressor was first demonstrated in murine epidermal JB6 cells. JB6 P+ cells are tumorigenic and have low levels of PDCD4, whereas JB6 P- cells are not tumorigenic and express PDCD4. PDCD4 knockdown by antisense transfection of P- cells rendered the cells susceptible to transformation, whereas overexpression of the recombinant protein made transformation sensitive P+ cells insensitive to tumour promotion (6, 22, 23). Overexpression also suppresses the maintenance of the tumour phenotype in RT101 (permanently transformed JB6 P+) cells (24). Subsequently, PDCD4 was found to inhibit the malignant phenotype in ovarian cancer cells (11) and suppress malignant metastasis (25, 26), with loss of the protein associated with increased invasion (27, 28). Conversely, increased PDCD4 expression inhibits invasion by upregulating Tissue Inhibitor of Metalloproteinase 2 (TIMP-2) expression, inhibiting metaloproteinase (MMP) degradation of the basement membrane (25). Decreased invasion after an increase in PDCD4 expression has been observed in colorectal cancer, which was associated with diminished Urokinase-type plasminogen activator receptor (uPAR) expression (29). PDCD4 expression is therefore generally associated with tumorigenesis, tumour progression and metastasis.

## PDCD4 Binds to eIF4A and Inhibits Protein Translation

Subsequent to the discovery that PDCD4 acts as a tumour suppressor in JB6 cells, the protein was found to inhibit translation and bound the eukaryotic translation-initiation factor 4A (eIF4A) (30). Further investigations showed that PDCD4 suppresses cap-dependent translation by competing with eIF4G for the binding of eIF4A, preventing the formation of a functional translation complex (31, 32).

It has been proposed that translational inhibition by PDCD4 is discriminatory and the decreased expression of specific proteins results in the suppression of tumorigenesis. One such down-regulated protein is carbonic anhydrase (CA) II, which is required for a high bicarbonate flux needed metabolically for proliferation in some carcinoid cells and decreased translation of CAII in Bon-1 and HEK293 cells by PDCD4 inhibits cell growth (33, 34). The expression of CAII has been found to correlate with biological aggressiveness in colorectal cancer (35), although this has yet to be associated with PDCD4 suppression. However, while there is frequently a direct inverse correlation between PDCD4 expression and proliferation, it is unclear whether the tumour suppressor ability of PDCD4 is solely due to the inhibition of translation by the protein, as few specific proteins that are direct targets of PDCD4 translational suppression and that influence cellular proliferation have been identified.

As well as the potential inhibitory action on translation in the cytoplasm, PDCD4 is also found in the nucleus and the role of the protein is likely to be dependent on its location. The protein has two nuclear translocation sequences and it has been proposed that phosphorylation may determine the location of PDCD4 and that different phosphorylation states occur in the nucleus and cytoplasm (20, 22, 36). PDCD4 has been found predominantly in the cytoplasm of normal breast tissue, though in the nucleus in breast ductal carcinoma (9, 10), suggesting that location dictates function with PDCD4 acting as a tumour suppressor by inhibiting CAP -dependent translation in the cytoplasm in normal cells. However, a similar study also examining the transition of normal breast tissue to invasive ductal breast cancer (IDC) has shown opposing findings, with cytoplasmic staining for PDCD4 being increased from normal cells to IDC and a concomitant decrease in nuclear staining (10). It is unclear why there is disparity between these two studies, but in corroboration with the latter study, cytoplasmic translocation from the nucleus during tumorigenesis has also been seen in the transformation of normal colorectal cells into adenomas and tumours, which was also associated with a loss of expression (16). Loss of nuclear expression in colorectal cancers has also been suggested as a prognostic indicator for poor survival (37).

## Relationship Between PDCD4 and Proliferation, Differentiation and Senescence

PDCD4 has been labelled a tumour suppressor in part due to the inverse relationship between expression and cell proliferation. Expression of the tumour suppressor protein is associated with an increase in p21 Cyclin-dependent kinase-interacting protein 1 (p21^cip^), which inhibits Cyclin-dependent kinase (CDK)4/6 and CDK2 (38, 39) and results in decreased proliferation, with a greater percentage of cells being in the G0/G1 phase of the cell cycle (40). This indicates that PDCD4 may promotes quiescence or senescence in cells. However, there is not an inverse relationship between proliferation and PDCD4 expression in all cells, as lymphoma cells treated with topoisomerase inhibitors decreases both the expression of the protein and mitogenesis (2), although this suppression of proliferation might be due to pathways other than that of PDCD4.

The relationship between PDCD4 expression and differentiation seems to be complex, with reports showing either a positive or negative correlation. In an inverse relationship, PDCD4 expression was found to be increased with diminished ovarian cancer differentiation (11), while treatment of human smooth muscle cells with bone morphogenetic protein 4 (BMP-4) and transforming growth factor beta (TGF-β) was found to decrease PDCD4 expression during smooth muscle differentiation (41). However, positive correlations have also been reported, with decreased PDCD4 expression being associated with impaired differentiation in nasopharyngeal carcinoma (7) and pancreatic cancer (42). Furthermore, transgenic mice overexpressing PDCD4 in the dermis have decreased proliferation and increased cellular differentiation associated with catagen entry (43), whereas PDCD4 translocation to the nucleus of cutaneous cells is also associated with differentiation, but not proliferation (44). PDCD4 is also upregulated during the inhibition of epithelial to mesenchyme transition (EMT) in breast cancer cells (40), a process which can be viewed as dedifferentiation (45). Overall, the relationship between expression and differentiation is likely to be cell type and signalling pathway specific (46).

As well as an association with proliferation and differentiation, PDCD4 up-regulation has also been linked with senescence in human diploid fibroblasts (47), with serum withdrawal resulting in decreased PDCD4 expression in T98G glioblastoma cells (48) and translocation from the nucleus to the cytoplasm in NIH 3T3 fibroblasts (49).

## PDCD4 and Apoptosis

As the name suggests, PDCD4 is associated with increased apoptosis, originally in thymocytes, T- and B cells and pheochromocytoma cells (3). The protein was subsequently found to be upregulated during apoptosis in fragmenting human embryos (50) and in breast cancer cells after treatment with the retinoic acid receptor (RAR) agonist, Ferentimide, which increases apoptosis, whilst decreasing proliferation (51). In addition, TGF-β1 treatment of Huh7 hepatocellular carcinoma cells resulted in increased PDCD4 and programmed cell death (52). The causative role of PDCD4 in Huh 7 cell apoptosis was seen by the overexpression of the protein, which induced programmed cell death, whereas cells that had reduced PDCD4 expression by anti-sense knock-down were resistant to TGF-β-induced apoptosis. The role of PDCD4 during apoptosis is further exemplified in glioblastoma cells that overexpress miRNA-21, a microRNA which inhibits PDCD4 translation and also impedes PDCD4- dependent apoptosis (53).

The ability of PDCD4 to induce apoptosis has been exploited during aerosol delivery of the protein to mouse lungs, resulting in decreased proliferation and increased programmed cell death, due to, at least in part, suppression of activator protein 1 (AP-1) activity (54, 55).

## The Interactions of PDCD4

Transfection and overexpression of PDCD4 suppresses the tumour phenotype in RT101 (JB6) epidermal cells and inhibits AP-1-dependent transcription needed for this phenotype (23, 56, 57). However, AP1 activation, although essential, is not sufficient for the conversion of JB6- (insensitive to tumour promotion) to JB6+ phenotype, which are sensitive to tumour promotion (58), indicating that PDCD4 must modulate an additional pathway other than AP-1 regulation in these cells to suppress tumorigenesis. In other cell types, decreased PDCD4 expression in HT29 colon carcinoma cells is associated with an increase in β catenin/T-cell factor (TCF)- and AP-1-dependent transcription (28), whereas increased exogenous PDCD4 in lung results in decreased AP-1 and increased apoptosis (54).

Inhibition of AP-1 activity by PDCD4 is due to blocking c-Jun phosphorylation, although it was initially thought that this was not by general suppression of c-Jun N-terminal kinase (JNK) activity (59). However, other studies have found that PDCD4 decreases the expression of mitogen-activated protein kinase 1 (MAP4K1), an upstream kinase in the JNK pathway, which consequently results in reduced JNK and cJun phosphorylation (57). Furthermore, phosphorylation of PDCD4 at Ser 67 and Ser 457 by protein kinase B (PKB/Akt) is associated with translocation to the nucleus (see below) and a decreased ability of PDCD4 to interfere with AP-1 response element transactivation (36). PKB activation can therefore increases AP-1 activity by downregulating PDCD4 and enhancing MAP4K1 and JNK activity and cJun phosphorylation.

AP-1 activation is required for transcription of miRNA21, a microRNA found to inhibit PDCD4 translation. Cytoplasmic PDCD4 can suppress AP-1 activity, resulting in decreased miRNA21 and the abrogation of PDCD4 inhibition by this microRNA (60). Therefore, PDCD4 may inhibit its own degradation by miRNA21 in a feedback loop.

It has been proposed that phosphorylation by PKB induces translocation of PDCD4 to the nucleus and decreases the ability of the protein to modulate the activity of AP-1 in NIH-3T3 cells (36). This is in direct contrast to evidence of PDCD4 translocation to the cytoplasm after PKB phosphorylation in resected colorectal cancer (16). NIH-3T3 cells are an immortalised transformed cell line and consequently have a corrupted cell cycle and these differences in findings may exemplify dissimilarities between *in vivo* and *in vitro* expression, with the tissue milieu influencing the functionality of the protein. Although PKB phosphorylation of PDCD4 has been shown not to interfere with eIF4A binding or inhibition of translation, a process that occurs in the cytoplasm (61), the relevance of this is unclear should PKB induce PDCD4 to translocate to the nucleus.

PDCD4 may be degraded after PKB and p70 ribosomal s6 kinase (p70 S6K) phosphorylation, which targets the protein for ubiquitination and proteosomic destruction. Both protein kinase C (PKC)-dependent activation of the phosphatidylinositol 3-kinase (PI3-K)/PKB/mammalian target of rapamycin (mTOR)/p70 S6K and extracellular signal-regulated kinase (ERK) signalling pathway are needed for this process, which discriminates between PKB phosphorylation causing PDCD4 nuclear translocation or degradation. Ubiquination and degradation occurs after stimulation with mitogens, as seen after the addition of the phorbol ester TPA to mouse skin papilloma cells, keratinocytes and human HEK293 cells, and was also observed in HEK293 cells after the addition of FBS (63). In addition, breakpoint cluster region protein- (BCR)-ABL transformed leukemic cells have a constitutively active mTOR/p70 S6K pathway, suppressed PDCD4 expression and a high proliferative rate. Inhibition of the BCR-ABL kinase by imatinib and mesylate suppresses PDCD4 degradation, increases apoptosis and decreases proliferation (64). Similarly, the anti-tumorigenic effects of statins may be due to decreased p70 S6K activation and the subsequent maintenance of higher levels of PDCD4. This results in increased apoptosis and decreased proliferation in renal cell carcinoma (65).

As PDCD4 has been shown to be downregulated after stimulation with mitogens, abrogating the possible anti-proliferation and apoptosis-inducing function of the protein, it may be questioned how PDCD4 acts as a tumour suppressor. However, fibroblasts transfected with non-degradable PDCD4 were found to be smaller and had a slowed cell cycle (48). Exactly how this occurs remains to be found.

Although substantial work has been completed on the relationship between PDCD4 expression and the interaction of the protein with cytoplasmic translation complex, little is known about interactions within the nucleus. However, it was found that PDCD4 directly interacts with the basic helix-loop-helix (bHLH) transcription factor Twist (66), a protein which has been attributed to having a role in mesoderm formation, myogenesis, neurogenesis and neural crest cell migration and differentiation (67), as well as endowing stem cell characteristics in tumour cells and promoting EMT (68). Twist has increased expression in oesophageal squamous cell carcinomas (69), is associated with gastric cancer progression (70) and increased proliferation and invasion in CRC (71). PDCD4 binding to twist results in the down regulation of the Twist target, Y-Box binding protein 1 (TB-1) and consequently a reduction in cell growth (66). Therefore, PDCD4 may directly affect gene transcription, although the precise mechanism of action is still unknown.

## PDCD4, the Immune System and Inflammation

PDCD4 expression can be influenced by the activation of the immune system and may influence inflammation. PDCD4 expression has been found to be modulated by differential immune activation in natural killer (NK) and T-cells, with interleukin-2 (IL-2) and IL-15 causing a reduction in expression, whereas IL-12 increases- and IL-4 and IL-7 maintains PDCD4 expression (72). However, PDCD4 is considered as pro-inflammatory. Although PDCD4 deficient mice have activated lymphocytes that produce cytokines that promote oncogenesis and develop spontaneous lymphomas which frequently metastasise, these mice were found to be resistant to inflammatory disease such as autoimmune encephalomyelitis (73) and diabetes (74). PDCD4 deficient mice also have decreased inflammatory cytokine production (75), with anti-inflammatory IL-10 production being increased in macrophages (76) and PBMCs (77). In addition, decorin, a soluble extracellular matrix proteoglycan and early response gene associated with inflammation and sepsis, increases PDCD4 expression in macrophages and decreases the release of anti-inflammatory IL-10 (78). The suppression of inflammation has been associated with the upregulation of miRNA-21 (77, 79, 80) and downregulation of PDCD4. miRNA-21 is a microRNA which binds to the 3’UTR of PDCD4 mRNA, inhibits translation and targets the mRNA for destruction. However, although miRNA-21 can suppress inflammation and inhibit PDCD4 expression, these may be independent processes and not linked. As PDCD4 might be pro-inflammatory, questions therefore arise regarding the function of PDCD4 as a tumour suppressor during inflammation, a state that promotes neoplastic transformation, particularly in cancers of the GI tract.

Inflammation is associated with many cancers (81–83) and has been included as a hallmark of cancer development (84). Chronic inflammation in the GI tract has a strong link with oesophageal- (85, 86), gastric- (87) and colorectal cancers (88, 89). However, there is a distinction in tumour aetiology and progression between inflammation induced tumorigenesis and tumour-induced inflammation. Approximately 20% of cancers are associated with persistent infections and its associated chronic inflammation (90). A notable example is helicobacter pylori-induced gastritis, where infection can result in activation of nuclear factor kappa B (NFκ-B) and AP-1, the transcription of oncogenes, the hyperproliferation of gastric cells (91) and gastric cancer (92, 93). Other environmental factors may instigate an inflammatory response such as gastroesophageal reflux disease, which results in oesophagitis and may progress to carcinogenesis. In addition, chronic intestinal inflammation found in patients with inflammatory bowel disease (IBD) or Crohn’s disease increases the risk of colitis-associated cancer (CAC) (94) due to the disruption of the tumour microenvironment (73) and the initiation of tumour formation (64). However, only 2% of CRCs are preceded by inflammation such as IBD, although sporadic CRC is still strongly linked with immune cell infiltration and the secretion of inflammatory cytokines (94). These inflammatory cytokines may instigate tumorigenesis (95), in particular IL-6, IL-11, IL-17 and TNFα, have been found to cause GI cancers (96, 97).

In sporadic CRC, cells progress to adenomas by the initial loss of the tumour suppressor adenomatous polyposis coli (APC) (98), which is associated with tumour-elicited inflammation, the disruption of the integrity of the epithelium and adenoma growth (67). Inflammation, in this case, is tumour derived and further stimulates proliferation in tumour progenitors.

The association between the PDCD4 expression, inflammation and GI tract carcinogenesis is confounding. The inflammatory secretome of macrophages has been found to suppress PDCD4 expression in cancer cells such as RKO rectal carcinoma cells (99), indicating that PDCD4 expression is regulated by inflammation. This suppression is also seen in dextran sodium sulphate (DSS) induced colitis in mice, where inflammation decreases PDCD4 protein expression (99). In addition, pharmacological downregulation of cyclooxygenase-2 (cox-2) activity in HCA-7 human colonic adenocarcinoma cell line resulted in an increase in PDCD4 protein-, but not mRNA expression, again implying that PDCD4 is controlled by inflammation (100). However, knockdown of PDCD4 can cause a decrease in inflammatory markers such as tumour necrosis factor alpha (TNFa) and IL-1b (101) in macrophages and other non-immune cells (102), showing PDCD4 to be pro-inflammatory.

In mice with DSS induced colitis, PDCD4 knockdown resulted in increased secretion of inflammatory cytokines, such as IL-6, the subsequent induction of STAT-3 expression, with an increase in epithelial cell proliferation and progression to CRC, illustrating an anti-inflammatory and tumour repressor role for PDCD4 (103). Corroborating a possible inverse association between PDCD4 and inflammation, inducible overexpression of the miRNA-21, a micro-RNA that suppresses translation of the tumour suppressor, results in diminished expression of PDCD4, intestinal inflammation and CRC in transgenic zebrafish (104). However, further investigation is required to prove a cause-and-effect relationship between PDCD4 expression and inflammation in this model.

## The Regulation of PDCD4 Expression

The cellular levels of PDCD4 protein are regulated by the modulation of gene transcription (4, 105), proteasomal degradation mediated by PKB or p70 RS6K phosphorylation followed by ubiquitination (62) and inhibition of translation controlled by the expression of microRNAs (miRNAs). miRNAs may act either by directly binding and inhibiting the functionality of PDCD4 mRNA or indirectly by inhibiting the translation of upstream proteins that are essential for PDCD4 expression. This is highly dependent on the cell type, the degree of transformation and the subsequent microRNA population expressed within these cells.

MicroRNAs are small RNAs, containing about 22 nucleotides, that regulate the stability and therefore the translational efficiency of complementary target mRNAs and are thought to be important in tumorigenesis (106, 107) (see below). It has been found that expression of PDCD4 is post-transcriptionally down-regulated by numerous miRNAs, with the relationship between miRNA-21 being most extensively studied due to the strong association of this microRNA with oncogenesis (106). This regulation by miRNA-21 has been found to occur in a multitude of cell types, including those of colorectal cancer (108, 109), oesophageal squamous cell carcinoma (110), in glioblastoma cells (111), NK cell lymphomas/leukaemias (112), vascular smooth muscle cells (113), cardiac myocytes (111), breast cancer cells (114, 115), epidermal cells (115) and in some (116, 117), but not all (10), human breast tumours. Inhibition of miRNA-21 results in the up-regulation of PDCD4 and tissue inhibitor of metalloproteinase 3 (TIMP3), a protein which inhibits metalloproteinases (MMPs) and invasion in cholangiocarcinomas (109). In prostate cancer cell lines, down-regulation of miRNA-21 activity by antisense oligonucleotides also results in an increase in PDCD4 expression, but without a change in proliferation (118), possibly indicating that PDCD4 does not act as a tumour growth repressor in some cell types. Furthermore, Qi et al. found no correlation between miRNA-21 and PDCD4 expression in breast cancer cells (10), implying that PDCD4 expression is likely to be regulated by mechanisms overriding that of any possible post-transcriptional modification. This is in direct opposition to oestradiol-induced decreases in miRNA-21, which results in increased PDCD4 expression in immortalised MCF-7 breast cancer cells (119). These conflicting results indicate possible differences in PDCD4 regulation by miRNAs in immortalised cell lines and primary patient tissue samples and that caution should be employed when using miRNA expression as a surrogate for PDCD4 protein availability and tumour suppression.

It has been proposed that activation of the ERK pathway by receptor tyrosine kinases, such as human epidermal growth factor receptor 2 (HER2/neu) increases miRNA-21 expression and downregulates PDCD4 in breast cancer cells (27). An inverse relationship between ERK activation and PDCD4 expression is also seen in skin, where the activation of the cyclooxygenase pathway results in the formation of ()-13-hydroxy-10-oxo-trans-11-octadecenoic acid (13HOA), a suppressor of inflammation, a decrease in ERK and PI3K activation and an increase in PDCD4 expression. This was associated with decreased proliferation (120).

## PDCD4, MicroRNA and the Gastrointestinal Tract

MicroRNAs are 20–25 nucleotides in length and usually bind to a complimentary sequence in the 3’-untranslated region (UTR) of target mRNAs, resulting in the degradation of the target and the downregulation of translational protein synthesis (121). Bioinformatic analyses (122) of the 3’-UTR of PDCD4 predict that this is a target for more than 80 miRNAs. As miRNAs may act in concert, with expression orchestrated by both genetic and environmental factors which ultimately determine the cells response and biological fate, the coordinated regulation of protein expression by miRNAs must therefore be complex. Direct overarching relationships between the expression of specific miRNAs and PDCD4 seen in published manuscripts should be viewed in the context of what would naturally occur in the cellular milieu *in vivo*. Coordinated expression of multiple miRNAs would be finely tuned to elicit a specific cellular response, of which, PDCD4 expression is likely to constitute just a small part.

A change in the expression of a number of miRNAs has been associated with cancers of the GI tract (123). Generally, from a review of the literature, investigators may use arrays or new generation sequencing to assess which miRNAs have been up- or down-regulated in tumours compared to adjacent normal tissue from patients and any differential expression is then confirmed by quantitative real-time PCR. Alternatively, cancer cell lines might be compared to immortalised lines of non-cancerous tissue. Single miRNA species which show major differential expression may then been chosen for further examination. Mimics (RNA duplexes that faithfully mimic the functions of mature miRNAs) or inhibitors (complementary chemically modified oligonucleotides) (124) of these miRNAs can be individually expressed in cell lines to establish whether the miRNA has effects on proliferation, apoptosis, cell migration and invasion. Mimics of miRNAs that are upregulated in GI tract cancers are usually found to increase proliferation, decrease apoptosis and promote cell migration and invasion, whereas the inhibitors have the opposite effects. Both PDCD4 mRNA and protein are now more frequently measured in cancers of the GI tract (**Table 1**) and compared to miRNA expression, as exemplified by the investigations subsequently outlined in this review, often with an inverse correlation showing a possible molecular relationship between them. The ability of the miRNA to directly influence PDCD4 expression can be initially assessed in silico using algorithms to predict whether the 3’-UTR of PDCD4 contains the binding site for the miRNA. By using luciferase assays, where the 3’-UTR of PDCD4 has been inserted into a reporter vector transfected into a cell line, direct binding can be monitored by changes in fluorescence elicited from the vector, with suppression seen as a decrease in fluorescence. Confirmation of this interaction is achieved by mutating the miRNA binding site on the 3’UTR of PDCD4, which should abolish suppression and not alter fluorescence.

**Table 1:** MicroRNA and PDCD4 expression in cancers of the gastrointestinal tract.

miRNA (Reference)	Tissue	Tissue *PDCD4* mRNA	Tissue PDCD4 protein	Cell lines used in study
miRNA-17 (154)	GC	↓	↓	MKN-45, AGS, SGC
miRNA-23 (156)	GC	↔	↓	MKN-45, AGS
miRNA-93 (155)	GC	↔	↓	AGS
miRNA-141 (198)	CRC	↓	NA	HT-29/oxa, HTP-116/oxa
miRNA-145 (199)	CRC	NA	NA	HT-29
miRNA-181 (200)	CRC	↔	↓	SW-480, CaCo2, HT-29
miRNA-183 (201)	GC	NA	NA	SGC-7901
miRNA-196a2 (202, 205)	CRC	↓	NA	HT-29, HCT-116
miRNA-208a (207)	GC	↔	↓	MKN-45, AGS, HGC-27
miRNA-320 (209)	ESCC	NA	NA	KYSE30, KYSE150, TE-1, EC109, EC9706
miRNA-320 (211)	GC	↓	NA	AGS, MGC-803, SGC-7901, SNU-1
miRNA-424 (241)	CRC	NA	NA	HCT-116
miRNA-499 (249)	CRC	NA	NA	SW-480, SW620
miRNA-503 (259)	CRC	↓	NA	SW-480, SW-620, HT-29, HCT-116
miRNA-503 (262)	CRC	Not specified	Not specified	SW-480, HCT-116
miRNA-1260b (264)	CRC	NA	NA	SW-480, HCT-116

CRC, Colorectal cancer; ESCC, Esophageal squamous cell carcinoma; GC, Gastric Cancer; ↓, downregulated; ↔, unchanged; NA, Not applicable/not measured.

To confirm the relationship between neoplastic transformation and PDCD4 expression, a vector overexpressing PDCD4 may be transfected into tissue-specific transformed cell lines to increase the expression of the protein, followed by the examination of changes in cell function, such as proliferation, apoptosis, cell migration and invasion. Similarly, miRNA mimics may be transfected into these immortalised cells followed by an assessment of PDCD4 expression and cell function to show that a specific miRNA can modulate tumorigenesis, possibly via its effects on PDCD4. These cells may also be transplanted into Severe Combined Immune Deficiency (SCID) mice, which have a compromised immune system and do not reject the xenotransplant, and the resultant tumours compared in size and mass to those from animals transplanted with non-transfected cells. Differences illustrate whether the miRNA is oncogenic (an oncomir) and if PDCD4 is anti-tumorigenic. Although the use of these methods indicates whether a specific miRNA influences PDCD4 expression in an immortalised cell line *in vitro* and *in vivo*, it is dependent on the cell line used, which might be somewhat different from primary cells. How the expression of these specific miRNAs is orchestrated within tumours *in vivo* remains a fundamental question that needs to be addressed to tailor chemotherapy in the future.

## The Relationship Between Specific microRNAs and PDCD4 Expression

### miRNA-21

The clinical implications of miRNA-21 expression have been extensively studies in a multitude of cancers and transformed cells (125, 126) including oesophageal- (127), gastric- (128) and colorectal cancer (129, 130). Originally, transfection of an miRNA-21 mimic and a luciferase-PDCD4 reporter construct into Colo206f colon adenocarcinoma cells showed decreased fluorescence and direct binding of miRNA-21 to the 3’-UTR of PDCD4. Overexpression of full-length PDCD4 reduce the ability of these cells for invasion, intravasation and metastasis (108). This has also been seen in immortalised gastric cancer cell lines, where overexpression of miRNA-21 resulted in decreased expression of PDCD4 and increased migration and invasion (131). A comparison between tumours and adjacent normal tissue has shown there is an inverse correlation between miRNA-21- and PDCD4 expression in GI cancers. PDCD4 protein is located in the nucleus of oesophageal epithelium (12, 13), normal gastric mucosa (132) and normal colocytes (12, 133) with limited expression in the cytoplasm. There is a progressive loss of nuclear PDCD4 from squamous intraepithelial neoplasia to squamous intramucosal carcinoma (12), with the progression of Barrett’s mucosa to Barrett’s adenocarcinoma (132) and from hyperplastic polyps to dysplasia, low- and high-grade adenoma and invasive CRC (133). This decreased expression is correlated with progression-free survival and overall survival in oesophageal cancer (134), gastric cancer (15) and Duke’s stage B, C and D CRC (135). Given the low levels of PDCD4 found in the cytoplasm in normal cells and a loss of the protein in the nucleus being associated with tumorigenesis, it is questioned how PDCD4 acts as a tumour suppressor in normal cells, as it is proposed that the major function of the protein is to inhibit translation in the cytoplasm (136). In addition, there are conflicting reports where elevated miRNA-21 and suppressed PDCD4 are not associated with staging or lymph node metastasis in oesophageal cancers (137) or tumour differentiation, lymph node metastasis or overall survival in gastric cancer (138). In the latter investigation, it was found that approximately 36% of the PDCD4 promotor was hypermethylated (138), suggesting transcriptional regulation rather than translational suppression of PDCD4.

The suppression of PDCD4 by miRNA-21 may be influenced by the secretome of adjacent cells in the tumour microenvironment (TME). Tumour cells can export miRNA-21 in exosomes, which fuse with the cell membrane of recipient cells, liberating the contents into the cytoplasm. This has been found to enhance cell migration and invasion in oesophageal cancer cells (139) and colon cancer cells (140). Exosomes containing miRNA-21 may also be derived from non-tumour cells located in the TME, such as resident fibroblasts (141) and macrophages (99). The miRNA-21-induced decrease in PDCD4 can result in increased chemoresistance to cisplatin in oesophageal cancer cells (141, 142) and to fluorouracil (5-FU) in colon cancer cells (140).

An increase in miRNA-21 expression and PDCD4 suppression during neoplastic transformation in GI cancers has been associated with an inflammatory response. Superoxide dismutase (SOD), catalase and total antioxidation capacity (T-AOC) are decreased, whereas 8-Oxo-deoxyguanosine (8-Oxo-dG), a marker of inflammation, is increased with elevated miRNA-21- and decreased PDCD4 expression and correlates with tumour staging, lymph node metastasis and remote metastasis in gastric cancer (143). In addition, MiRNA-21^-/-^ knockout mice, given azoxymethane and dextran sodium sulphate (DSS) to induce colitis-associated CRC, had decreased inflammatory cytokines, reduced tumour size and tumour number and increased PDCD4 expression compared to control wild-type animals (144). Furthermore, zinc deficiency is associated with inflammation, increased miRNA-21 expression, the suppression of PDCD4 and an increased risk of esophageal squamous cell carcinoma (ESCC) (145), whereas, the upregulation of COX2- (146) and miRNA-21- (147) and the decrease in PDCD4 (135) expression correlates with worse Duke’s stage. Also, the inhibition of COX-2 in HCA-7 colonic carcinoma cells results in decreased miRNA-21 and increases PDCD4, whereas treatment with prostaglandin E2 (PGE2) has the opposite effect (100). Although miRNA-21 expression negatively regulates pro-inflammatory response in haematopoietic cells, such as macrophages, it has been found to be pro-inflammatory in non-haematopoietic cells which may lead to neoplastic transformation (148). Tumours of the GI tract are frequently associated with inflammation, increased miRNA-21 expression and suppression of PDCD4. The inflammatory role of miRNA-21 in haematopoietic cells in the TME (149) is therefore questionable, but secretion of this microRNA by macrophages in exosomes (99) during immune dysregulation could directly impact on the transformation of recipient endothelial cells.

### miRNA-17-5p

miRNA17-5p has been found to be upregulated in oesophageal- (150), gastric- (151) and colorectal cancers (152) and although it may act as tumour suppressor in other tissues, it has been proposed that circulating miRNA-17-5p may be used for early cancer diagnosis (153).

The investigation by Zhao et al. (154) showed that increased expression of miRNA-17-5p in gastric cancer patients was associated with decreased PDCD4 mRNA and worse overall survival. Overexpressing miRNA-17-5p in MKN-45 gastric cancer cells increased proliferation and migration, and decreased PDCD4 expression, whereas silencing miRNA-17-5p had the opposite effect. A luciferase reporter assay confirmed direct binding of miRNA-17-5p to the 3’-UTR of PDCD4. Hypoxia-induced factor 1A (HIF1A) was found to be upregulated with miRNA-17-5p in GC cells and regulated the expression of the microRNA by directly binding to the miRNA-17-5p gene promotor. However, PDCD4 overexpression resulted in the downregulation of miRNA-17-5p, which is surprising as PDCD4 promotes HIF-1A expression and should consequently upregulate miRNA-17-5p. The authors suggest that further investigation is required to delineate the relationship between HIF-1A, miRNA-17-5p and PDCD4 expression.

### miRNA-23a/b

Arrays have been utilised to identify miRNA23a/b upregulation in gastric cancer (155) and Hu X et al. (156) subsequently had corroborative findings in 10 gastric cancers when compared to adjacent, non-cancerous tissue. An inverse correlation was also found between miRNA23a/b and PDCD4 protein, but not with PDCD4 mRNA levels, inferring that miRNA23a/b might inhibit PDCD4 translation, but does not result in mRNA degradation. This was also confirmed in MKN-45 and AGS human gastric cancer cell lines. Direct interaction between miRNA-23a/b and PDCD4 was seen by the overexpression a miRNA23a/b mimic and PDCD4 3’-UTR reporter. Knockdown of PDCD4 in MKN-45 cells resulted in decreased apoptosis, whereas overexpression increased programmed cell death, confirming the role of the tumour suppressor as being pro-apoptotic. Furthermore, transfection of miRNA-23a/b mimic into these cells resulted in decreased apoptosis, whereas antisense miRNA-23a/b Antagomir increased programmed cell death, possibly by the modulation of PDCD4 expression. To test this, co-transfection of a miRNA-23a/b-sensitive PDCD4 overexpression plasmid with a miRNA-23a/b mimic resulted in increased PDCD4 protein and elevated apoptosis, suggesting that PDCD4-induced apoptosis is directly modulated by miRNA-23a/b in MKN-45 cells. The potential for miRNA-23a/b to be an oncomir was confirmed by implanting MKN-45 cells overexpressing miRNA23a/b mimic into SCID mice. The tumours from these cells had decreased PDCD4 expression, increased mitosis, decreased apoptosis and elevated size and weight.

### miRNA-93

Increased expression of miRNA-93 has been seen in a wide variety of cancer types (157), with upregulation in oesophageal cancers (158, 159) being associated with poor prognosis and decreased efficacy of radiotherapy (160). *In vitro* forced overexpression in oesophageal cell lines has been found to increase proliferation, migration and invasion (159, 161) concomitant with a decrease in the direct targets of miRNA-93, transforming growth factor beta receptor 2 TGFβR2). (161) and disabled 2 (DAB2) (159). Similarly, this microRNA has been found to be upregulated in gastric cancer (162, 163) and expression correlates with poor survival and metastatic disease (164, 165), Transfection and overexpression of miRNA-93 in gastric cancer cell lines also promotes an increase in proliferation, migration, invasion and chemoresistance (166, 167) and decreases the expression of the tumour suppressors AHNAK (167, 168) and TIMP-2 (165). However, there is conflicting data regarding the expression and contribution of miRNA-93 in colorectal cancer, as expression has been found to be increased in CRC tissue (169), downregulated by chemotherapeutic drugs (170) and negatively regulates the expression of the tumour suppressor PTEN in SW620 cells (171), yet has also been found to be downregulated in CRC tissue (172–174), with suppression associated with metastatic disease and poor overall survival (175). In addition, other investigations have shown that overexpression of miRNA-93 in CRC cell lines decreases cell viability, increases apoptosis (176) and can suppress proliferation and colony formation (177). The reason for the disparity between investigations attributing miRNA-93 as a tumour suppressor and those assigning the microRNA as an oncomir in CRC, the latter in corroboration with its proposed role in oesophageal and gastric cancers, is confounding. This cannot be accounted for by inter-tumour variability as tissue from each cohort shows similar results which are statistically different from the control tissue.

In accord with other finding (162, 163), the study by Liang et al. (155) found that miRNA-93 was also a possible oncomir in gastric cancer cells. The investigators used a miRNA array to examine two gastric cancer tissues and found that miRNA-23b-3p, miRNA-17-5p and miRNA-93 inversely correlated with PDCD4 expression and were therefore candidate regulators of the tumour suppressor. Transfection of mimics of these miRNAs into gastric cancer AGS cells containing a PDCD4 3’ UTR reporter plasmid reduce fluorescence, indicating direct binding to the 3’ UTR and suppression of translation. Similar to the investigation examining miRNA-23a/b in MKN-45 cells (see above), the over-expression of miRNA-93 in AGS cells resulted in decreased PDCD4 protein expression, with no effect on PDCD4 mRNA levels, indicating an inhibition of mRNA function rather than increased degradation. This was concomitant with decreased apoptosis, whereas knock down of miRNA-93 increased PDCD4 expression and programmed cell death. Confirming the relationship between expression and apoptosis, PDCD4 knockdown decreased-, whereas overexpression increased programmed cell death. Transfection of miRNA-93-insensitive PDCD4 was found to alleviate the anti-apoptotic effect of miRNA93, suggesting that miRNA-93 inhibits apoptosis by directly binding to the 3’ UTR and decreasing PDCD4 protein expression. A xenograph of AGS gastric adenocarcinoma cells overexpressing miRNA-93 was found to produce tumours which were larger and heavier than those from control cells not overexpressing the micro-RNA, suggesting that miRNA-93 is an oncomir which does not require coordinated expression with other micro-RNAs to promote tumour expansion. This leads to the question of why is there simultaneous expression of several microRNAs that can individually downregulate PDCD4 in certain cancer cells and suggests it might be likely that the tumorigenic agent is upstream- and similarly effects expression of these miRNAs.

### miRNA-141

Predictions using the Cancer Genome Atlas (TCGA) show that miRNA-141 expression is associated with lymph node metastasis in patients with ESCC (178) and comparisons of ESCC tissue with adjacent normal tissue have shown expression to be associated with tumour differentiation status and chemoresistance (179). In ESCC cell lines, miRNA-141 silencing decreases cell proliferation, migration and invasion (180), whereas increased expression downregulates SOX17, thereby activating the Wnt signalling pathway (181), and/or pleckstrin homology domain leucine-rich repeat protein phosphatase-2 (PHLPP2) which subsequently increases the activity of Akt/PI3-K pathway (182). However, miRNA-141 has been found to be downregulated in gastric cancer tissue (183) and is inversely associated with tumour differentiation (184), lymph node- and distant metastasis and overall survival (185). The ability of miRNA-141 to suppress gastric tumours has been seen in GC cell lines where overexpression decreases proliferation, migration and invasion (185, 186) and xenotransplantation of GC cells that over-express miRNA-141 results in smaller tumours compared to control, non-transfected cells (187).

The contrast between miRNA-141 being an oncomir in ESCC cells and a tumour suppressor in gastric cells is mirrored in investigations examining this microRNA in colorectal cancer. In corroboration with evidence examining oesophageal cancer, where miRNA-141 was found to be an oncomir, some studies have found that miRNA-141 is elevated in CRC tissue (188–190), is positively associated with stage progression (189), increases proliferation in CRC cell lines (188–190) and results in larger tumours in xenotransplants of CRC cells overexpressing the microRNA (188). However, the opposite has also been found, with miRNA-141 being suppressed in CRC tissue (191, 192), with the level of suppression related to differentiation stage (193) and poor prognosis (191, 192). Further evidence that miRNA-141 is a possible tumour suppressor in CRC is shown by increased expression of the microRNA being associated with greater sensitivity to chemotherapy (194–196) and overexpression in CRC cell lines suppresses proliferation, migration and invasion (191, 194). Xenotransplantation into nude mice of CRC cells overexpressing miRNA-141 results in tumours which are smaller than those produced by control, non-transfected cells (191, 197).

Wang et al. examined the relationship between the expression of the long non-coding RNA (LncRNA) MEG3, miRNA-141 and PDCD4 and chemoresistance, as MEG3 was found to be suppressed in the oxaliplatin resistant tumours of CRC patients and is a potential target of miRNA-141 (198). MEG3 expression was shown to be lower in SW480, HT29 and HCT-116 CRC cell lines compared to normal NCM460 colon epithelial cells and suppressed further in oxaliplatin resistant HT29/OXA and HCT-116/OXA cell lines, indicating a relationship with chemo-resistance as well as tumorigenesis. Furthermore, CRC patients with lower levels of MEG3 had a poor prognosis and shorter overall survival. The relationship with chemo-resistance was further exemplified by transfection of MEG3 into both HT-29/OXA and HCT-166/OXA cell lines, which reduced chemo-resistance by enhancing oxaliplatin-induced apoptosis. As LncRNAs are known to modulate the expression and activity of microRNAs, predictive analysis was completed to determine which miRNAs bind to MEG3 and direct binding of miRNA-141 was confirmed by the use of luciferase assays, with overexpression of miRNA-141 resulting in reduced activity in the MEG3 promoter. Examining miRNA-141 expression in CRC tumours, it was found that there was an inverse relationship with the levels of MEG3 and oxaliplatin resistance. This was corroborated by transfecting HT29/OXA and HTP-116/OXA cells with miRNA-141 inhibitor, which reduced cell number by increasing oxaliplatin-induced apoptosis. PDCD4 was predicted as a target of miRNA-141, which was confirmed using luciferase reporter assays. Both PDCD4 mRNA and protein were suppressed when either miRNA-141 mimic or a MEG3 inhibitor were transfected into HT29/OXA or HTP-116/OXA cells. PDCD4 expression was also found to positively correlate with MEG3 expression in tumour cells. Transfection of PDCD4 resulted in increased oxaliplatin-induced apoptosis, which was abrogated by co-transfection with either miRNA-141 or a MEG3 inhibitor. It was concluded that the tumour suppressive effects of MEG3 were by sponging (binding and inactivating) miRNA-141 activity and expression, thereby preventing the microRNA from suppressing the expression of PDCD4.

### miRNA-145

Cancer stem cell enriched, chemo-resistant colon cancer has been found to have increased expression of miRNA-21 and decreased expression of miRNA-145. Yu Y et al. (199) compared chemo resistant (CR) HT-29 colon adenocarcinoma cells and parent control (chemo-sensitive) HT-116 cells and found that miRNA-145 was downregulated by 62%, whereas miRNA-21 was upregulated by 90% in the resistant cells. Xenotransplantation of these cells into SCID mice, showed the CR cells had a 40% reduction in PDCD4 expression. Injecting the mice transplanted with CR HT-29 cells after tumour formation with a complex of polyethylenimines (PEI)/miRNA-145 increased the miRNA-145 by 50%, suppressed tumour growth and increased the PDCD4 concentration significantly by 10%, demonstrating that miRNA-145 could partially abrogate the effects of chemo resistance in these cells.

Transfection and the expression of anti-miRNA-21 into CR HT-29 cells resulted in a six-fold increase in miRNA-145, indicating that miRNA-21 suppresses miRNA-145 expression, whereas transfection and overexpression of miRNA-145 in this cell type resulted in a 70% decrease in miRNA-21 expression, showing a reciprocal relationship, where miRNA-145 can suppress miRNA-21. Given that PDCD4 has a strong negative relationship with miRNA-21, it would have been informative to know if the polyethylenimines (PEI)/miRNA-145 treatment resulted in a decrease in miRNA-21, an oncomir with several targets other than PDCD4. Although the authors stated that there was a statistically significant effect on PDCD4 expression, this seems relatively minor (10%) and it is questioned whether this is significant biologically and whether suppression of tumour growth may have occurred *via* another tumour suppressor.

### miRNA-181b

Although miRNA-181b was upregulated and PDCD4 protein downregulated in CRC tissue compared to adjacent normal tissue, PDCD4 mRNA expression was found to be inconsistent (200). The overexpressed of miRNA-181b mimic in SW480 colon adenocarcinoma cells resulted in an increase in proliferation and migration, whereas apoptosis and PDCD4 protein expression decreased, with only a marginal decrease in mRNA. Conversely, knockdown of miRNA-181b resulted in the abrogation of proliferation and migration and an increase in both apoptosis and PDCD4 protein expression. Similar to SW480 cells, these changes in PDCD4 mRNA and protein expression were also seen in Caco2 and HT29 cells. PDCD4 expression was knocked down in SW480 cells resulting in an increase in both proliferation and migration and decreased apoptosis, while overexpression of PDCD4 had opposing effects. To examine a possible direct relationship between miRNA-181b and PDCD4, miRNA-181b-resistant PDCD4 was transfected into SW480 cells. This rescued PDCD4 suppression induced by miRNA-181b, resulting in a decrease in proliferation and migration and an increase in apoptosis. A xenotransplant of SW480 overexpressing miRNA-181b into SCID mice produced tumours with increased weight, size and cellular proliferation, which was rescued by overexpressing miRNA-181b-insensitive PDCD4. This showed a direct relationship between miRNA-181b and PDCD4 in SW480 cells, which had direct consequences on tumour progression *in vivo*.

### miRNA-183

Transfection of SGC-7901 gastric cancer cells with miRNA-183 mimic increased proliferation and invasion (wound healing assay), and a concomitant decrease in apoptosis. Furthermore, both PDCD4 mRNA and protein were down regulated. Conversely, transfection of the miRNA-183 inhibitor decreased proliferation and invasion and increased both apoptosis and PDCD4 protein expression (200, 201). Unfortunately, no cause-and-effect relationship between miRNA-183 and PDCD4 expression was shown and consequently further investigations are required to confirm this relationship.

### miRNA 196a2

The microRNA miRNA-196a2 has been found to be upregulated in gastrointestinal - (202), and oesophageal tumours (203) and although a common polymorphism in the miRNA-196a2 gene is associated with increased risk of colorectal and gastric cancer (204), the association between miRNA-196a2 and GC has yet to be proven at a cellular level.

Examining the relationship between miRNA-196a2 and CRC, Ye et al. (205) transfected a miRNA-196a2 mimic into HT-29 and HCT-116 cells. This increased proliferation and colony number, whereas apoptosis and PDCD4 protein expression were decreased. It was predicted that miRNA-196a2 is directly negatively regulated by the long intergenic non-coding RNA tumour suppressor Linc 00472. Concomitant overexpression of Linc 00472 was found to abrogate the effects of miRNA-196a2 overexpression. To examine whether PDCD4 expression and proliferation are linked and not incidental, PDCD4 expression was knocked down in these cells, which promoted proliferation and reduced apoptosis. The direct interactions between miRNA-196a2 and Linc 00472 and between miRNA-196a2 and PDCD4 were confirmed by utilising luciferase reporter assays after expressing the transcripts in 293T embryonic kidney cells. These cells were used, rather than immortalised CRC cells, as they can be robustly transfected with multiple transcripts. Transfection of Linc00472 reversed the miRNA-196a2 inhibition of PDCD4 expression, with Linc 00472 positively sponging miRNA-196a2. The converse was also found, where overexpression of miRNA-196b reversed the suppression of proliferation and the increase in PDCD4 and apoptosis propagated by Linc 00472. This indicates that there is likely to be a subtle balance between miRNA-196a2 and Linc 000472 in order to adequately regulate cellular proliferation. The effect of overexpressing Linc00472 in HT-29 cells *in vivo* was analysed by xenotransplantation into nude mice. These cells subsequently formed tumours that were smaller than the controls (formed by the injection non-transfected cells), had diminished miRNA-196a2 expression, decreased proliferation and increased PDCD4 expression. It was concluded that Linc 00472 could act as a tumour suppressor by sponging miRNA-196a2 and thereby increasing PDCD4 expression.

### miRNA-208a

MiRNA-208a has been found to promote proliferation in oesophageal squamous cell carcinoma (206) and is elevated in gastric cancers (207) and CRC (208). Examining gastric cancers, Yin et al. found an inverse relationship between miRNA-208a and PDCD4 protein expression, although PDCD4 mRNA was unchanged, indicating that protein suppression is likely to be by inhibition of translation and not mRNA degradation (207). Overexpressed and knocked down of miRNA-208a in MKN45, HGC-27 and AGS gastric cancer lines caused suppression- or increased expression of PDCD4 protein respectively. The predicted direct interaction between miRNA-208a and PDCD4 was confirmed using luciferase reporter assays where miRNA-208a was shown to directly bind to the 3’UTR of PDCD4.

PDCD4 expression was found to be related to apoptosis in MKN45 cells, with PDCD4 knockdown resulting in decreased-, whereas overexpression increased programmed cell death. The diminished apoptosis seen after miRNA-208a transfection was rescued by co-transfection with a plasmid that overexpresses PDCD4 insensitive to this microRNA, inferring that the anti-apoptotic action of miRNA-208a is mediated via PDCD4. In addition, MKN45 cells transfected with miRNA-208a and xenotransplanted into SKID mice resulted in tumours that were heavier and larger and had a higher proliferation index than transplanted untransfected control cells. However, co-transfection of miRNA-208a-insensive PDCD4 with miRNA-208a decreased the size and weight of the tumour, although total abrogation of tumour growth did not occur, even though apoptosis had increased. This indicates that although increased PDCD4 expression could retarded the process, other factors ultimately control cellular proliferation and tumour growth. The vast majority of these findings were confirmed in a highly similar manuscript examining CRC, where the investigators utilised HCT116, SW480, SW620, and HT-29 cell lines (208).

### miRNA-320b

Next generation sequencing used to analyse exosomes of oesophageal squamous cell carcinomas (ESCC) revealed that miRNA-320b was highly expressed in many ESCCs and correlates with lymph node metastasis (209). Exosomes purified from KYSE150 and EC9706 ESCC cell lines were found to be enriched with this miRNA and co-incubation of these exosomes with human lymphatic endothelial cells (HLECs) stimulated tube formation and migration, processes which occur in lymphangiogenesis during lymph node metastasis. The recipient HLEC cells treated with ESCC exosomes were also found to have significantly higher levels of phosphorylated AKT, a kinase also associated with lymphangiogenesis and lymphatic metastasis and suppressed PDCD4 mRNA and protein. PDCD4 expression has previously been associated with the suppression of the AKT signalling pathway and lymph node metastasis and computational analysis showed that PDCD4 could be a target of miRNA-320b. This was confirmed in HLEC cells as both exosome-induced tube formation and migration, as well as AKT phosphorylation, could be abrogated by the overexpression of PDCD4. Direct binding of miRNA-320 to the 3’-UTR of PDCD4 was shown in 293T embryonic kidney cells using luciferase assays and by mutating the miRNA binding site. METTL3 is a methyltransferase integral to the process of microRNA maturation and is associated with tumorigenesis (210). This methyltransferase was found to be upregulated in ESCC tissue and EC9706 cells. Overexpression of the METTL3 resulted in an increase in miRNA-320b, whereas knock down had the opposite effect. As xenotransplants of KYSE150 cells secreting exosomes containing miRNA-320b showed a greater number of metastatic lymph nodes, it was concluded that in ESCC, METTL3 increases miRNA-320b, which suppresses PDCD4 and activates the AKT pathway, increasing tumorigenic effects.

Similarly, Luo et al. (211) examined the relationship between miRNA-320b, PDCD4 and the long non-coding RNA (LncRNA) NR2F2-AS1 in gastric cancer. LncRNAs are non-protein coding RNAs greater than two hundred nucleotides in length that can bind and consequentially inactivate (sponge) microRNAs. Changes in the expression of LncRNAs have been associated with multiple cancers (212) including oesophageal- (213), gastric- (214) and colorectal cancers (215). The authors found that NR2F2-AS1 is weakly expressed in gastric cancer tissue and in AGS, MGC-803, SGC-7901 and SNU-1 GC cell lines compared to normal cells and expression negatively correlated with poor prognosis and worse overall survival. Overexpression of this LncRNA in AGS and MGC-803 cells inhibited proliferation, cell migration and invasion and increased apoptosis, illustrating the role of NR2F2-AS1 as a tumour suppressor. Computational analysis predicted binding of NR2F2-AS1 to miRNA-320b, which was confirmed *in vitro* by Luciferase reporter assays. An inverse correlation between NR2F2-AS1 and miRNA-320b expression in GC tissue and cell lines predicts sponging between the RNAs. Down regulation of miRNA-320b using a specific antagomir in AGS and MGC-803 cells reduced cell viability, migration and invasion and increased apoptosis. Exploring the relationship between miRNA-320b and PDCD4, luciferase assays showed that the microRNA directly binds to the 3’UTR of PDCD4, reducing fluorescence and overexpression of miRNA-320b resulted in a decrease in both PDCD4 mRNA and protein. PDCD4 was negatively associated with miRNA-320b and positively associated with NR2F2-AS1 in GC tissue. In addition, the antiproliferative and pro-apoptotic effects of overexpressing NR2F2-AS1 were diminished by overexpressing miRNA-320b or down-regulating PDCD4 expression. Overall, it was concluded that NR2F2-AS1 acts as a tumour suppressor in gastric cancer by sponging miRNA-320b, alleviating possible suppression of PDCD4 by this microRNA.

Unlike the investigation on gastric cancer outlined above, miRNA-320b has been found to be repressed in CRC tissue (216, 217) and LoVo, SW620, HCT116 and SE480 CRC cell lines (218) compared to normal issue and normal HIEC cells, and overexpression of a miRNA-320b mimic was able to decrease proliferation, cell migration and invasion and promote apoptosis (219). This has also been found in other cancers (220–222) and miRNA-320b has been proposed as a tumour repressor that can be used as a biomarker for tumour progression (223). However, contrary to these findings, examination of 126 tissue samples found miRNA-320a to be upregulated in CRC (224). The reason for this disparity between investigations is unknown and as of yet, there are no investigations that have examined the relationship between PDCD4 expression and miRNA-320b in CRC.

### miRNA-424

Investigations examining the expression and function of miRNA-424 in cancers of the GI tract have shown the microRNA to either be a tumour suppressor or an oncomir. The differences between the findings are perplexing, with disparate data emanating from studies examining both tissue samples and cell lines. miRNA-424 has been found to be upregulated in squamous tissue from Barrett’s oesophagus (225) and oesophageal cancer tissue (226) and has been correlated with poor survival of ESCC patients (226). In addition, expression has been associated with increased proliferation and migration in ESCC cell lines (227). However, decreased expression has also been found in ESCC tissue, with suppression associated with increased lymph node metastasis (228). In ESCC EC-1 cells, increased expression of miRNA-424 decreases proliferation, migration and invasion and therefore the microRNA acts as a potential tumour suppressor (228). Expression of miRNA-424 in gastric tissue has also found to be either upregulated (229–231) or downregulated (232, 233), with some investigations finding suppression related to chemoresistance (232, 233). The disparity between miRNA-424 having properties of either an oncomir or tumour suppressor is also seen in GC cell lines (229, 230, 232, 234). Similarly, investigations examining miRNA-424 in colorectal cancer have found the microRNA to be either pro- (235–237) or anti-oncogenic (238–240).

Tumour cells under hypoxic conditions may become resistant to chemotherapy due to a decreased apoptotic response related to the induction of hypoxia induced factor 1A (HIF-1A). Consistent with this, hypoxia in immortalised HCT116 CRC-, U251 glioblastoma- and A375 melanoma cells was found to induce HIF-1a expression and the upregulation of miRNA-424 (241), a microRNA upregulated in CRC (242). In addition, the induction of HIF1a by hydrogen peroxide or dimethyloxalylglycine (DMOG), an inhibitor of HIF1a degradation, also resulted in elevated miRNA-424 levels. The direct relationship between HIF1a and miRNA-424 expression was shown by HIF-1A binding to the hypoxia response element (HRE) of pri-miRNA-424 using a chromatin immunoprecipitation (ChIP) assay. Overexpression of miRNA-424 resulted in the inhibition of doxorubicin-induced apoptosis indicating that miRNA-424 induced under hypoxic conditions might protect tumours from chemotherapy-induced programmed cell death. Investigating the mechanism by which this may occur, it was found that miRNA-424 can bind directly to the 3’-UTR of PDCD4 resulting in a decrease in both PDCD4 mRNA and protein levels in HCT116 cells. Knockdown of miRNA-424 in these cells resulted in a six times increase in PDCD4 mRNA and protein. Although indirect evidence strongly suggests that hypoxia results in increased HIF1a expression which subsequently upregulates miRNA-424 and in turn, downregulates PDCD4 expression and decreased apoptosis, a direct cause-and-effect relationship was not proved. This could have been rectified by overexpressing miRNA-424-insensitive PDCD4 in HCT116, subjecting the cells to hypoxia and measuring apoptosis.

### miRNA-499

There is a paucity of cellular and molecular studies examining the role of miRNA-499 in gastrointestinal cancers. However, it has been found that overexpression of the microRNA in EC9706 and KYSE30 ESCC cells decrease proliferation and colony formation and increase cisplatin-induced apoptosis, indicating that miRNA-499 is a tumour suppressor (243). The association of miRNA-499 with cancers of the GI tract has concentrated on examining the relationship between Single nucleotide polymorphisms (SNPs) in miRNA-499 rs3746444 and susceptibility to GI tract tumorigenesis. Investigators have found that specific polymorphisms can increase the risk of ESCC (244), GC (245) and CRC (246) or have no effect on GI cancer susceptibility (247, 248).

SW480 CRC cells are from an adenocarcinoma primary lesion, whereas highly metastatic SW620 CRC cells were taken from a metastatic lymph node of the same patient and consequently a comparison between these lines can be used as a model to study minimally- and highly metastatic CRC cells. Using arrays to analyse miRNA expression in these cell lines, miRNA-499-5p had the greatest fold difference (an increase of x8), a finding confirmed using qRT-PCR (249). Subsequent examination of CRC tissues from 90 patients revealed that miRNA-499-5p expression was associated with advanced clinical stage and lymph node metastatic disease. Transient expression of miRNA-499-5p mimic in SW480 cells increased migration in transwell and cell invasion assays, whereas transfection of a miRNA-499-5p inhibitor into SW620 cells impeded migration and invasion. However, stable transfection of miRNA-499-5p in SW480 cells did not induce tumour cell growth *in vitro*, but when injected into nude mice the number and size of the resultant metastatic nodules in the lung and liver were significantly more than vector controls. *In silico* investigation showed that both the tumour repressors Forkhead box protein O4 (FOXO4) and PDCD4 are candidate targets of miRNA-499-5p and direct binding of miRNA499-5p to 3’-UTR of the mRNAs was confirmed using luciferase assays. Both FOXO4 and PDCD4 mRNA and protein were found to be downregulated in highly metastatic SW620 cells and in SW480 cells stably transfected with miRNA-499-5p. Inhibition of miRNA-499-5p in SW620 cells resulted in an increase in FOXO4 and PDCD4. Examining CRC tissue, both FOXO4 and PDCD4 were found to be downregulated in lymph node positive-, compared to lymph node negative tissue. Knockdown of FOXO4 and PDCD4 in SW480 cells promoted migration, whereas knockdown of miRNA-499-5p reduced cell number. In SW620 cells, a miRNA-499-5p-inhibitor suppressed tumour cell migration. This could be only partially reversed by knockdown of FOXO4 or PDCD4 gene expression using siRNA, implying that migration is suppressed by FOXO4 and PDCD4 in concert.

### miRNA-503

miRNA-503 has been found to be highly expressed in ESCC and correlates with lymph node metastasis, TNM staging of the tumour (250) and poorer disease-free and overall survival (251). However, contrary to this, it has also been attributed to having tumour suppressor effects in ESCC (252–254) and other cell types (255). Similarly in gastric cancer, analysis of one thousand gastric cancer tissue samples showed miRNA-503 to be significantly upregulated (256), yet it has also been found to suppress GC cell growth and invasion (257) and EMT (258). In CRC, increased expression is also associated with migration, invasion (259), metastasis (237) and poor prognosis (260), although it has been shown to be down regulated in CRC tissue and linked to inhibition of proliferation and increased apoptosis (218). This disparity between the findings that miRNA-503 is either an oncomir or tumour suppressor highlights the complexity of cellular regulation by microRNAs. Examining the effects of individual miRNAs may discern the functionality of that microRNA, but this negates the orchestration of many miRNAs required to elicit and exquisitely regulate cellular responses *in vivo*. The limited investigations examining the relationship between miRNA-503 and PDCD4 have shown that this microRNA to be an oncomir in CRC.

CRC tumours and CRC cell lines (SW480, SW620, HT-29, HCT-116) were found to have increased expression of miRNA-503 and down-regulated PDCD4 mRNA when compared to healthy surrounding tissue and normal colon epithelial FHC cells (259). The prediction that the 3’-UTR of PDCD4 is a direct target of miRNA-503 was confirmed using luciferase assays and the modulation of PDCD4 expression by miRNA-503 was shown by transfection of either a miRNA-503 mimic or inhibitor, which resulted in decreased- or increased expression of PDCD4 respectively in both SW480 and HCT-116 cells. Transfection of the miRNA-503 mimic in SW480 cells also increased migration, whereas either the miRNA-503 inhibitor or the over-expression of PDCD4 retarded invasion. Overexpression of PDCD4 simultaneously with a miRNA-503 mimic resulted in decreased migration and invasion compared to the mimic alone and consequently the authors concluding that the pro-migratory and pro-invasion activity of miRNA-503 must be via PDCD4. However, although this assumption is highly plausible, evidence for a cause-and-effect relationship between miRNA-503 and PDCD4 expression was not given.

Circular RNAs are non-coding RNAs that have been associated with many cancers and may sponge and inactivate miRNAs (261). Wen et al. (262) examined the tumour suppressor effects of the circular RNA circ_0003266 and found it to be downregulated in CRC tissue and CRC cell lines (HT29, SW480, HCT-116, Lovo, and DLD-1) compared to normal colonic epithelial cells (NCM460). Overexpression of circ_0003266 in SW480 cells decreased cell growth, migration and invasion, but silencing in HCT-116 cells had the opposite effect. Overexpression also decreased miRNA-503-5p, whereas silencing increased the expression of this microRNA. Direct binding of circ_0003266 to miRNA-503-5p was seen by dual luciferase assays, which also showed direct binding of miRNA-503-5p to the 3’-UTR of PDCD4. Transfection of a miRNA-503-5p mimic was found to decreased PDCD4 mRNA and protein expression, whereas a miRNA-503 inhibitor had an opposing effect. The authors noted that increased expression of miRNA-503 in CRC tissue negatively correlated with PDCD4 expression, though it was not specified whether this was PDCD4 protein and/or mRNA. The expression of circ_0003266 inhibited proliferation, migration and invasion and promoted apoptosis in SW480 cells and increased PDCD4 mRNA and protein expression. This could be abrogated by the miRNA-503 mimic, which in turn could be rescued by overexpression of PDCD4. It was concluded that circ_0003266 can suppress CRC progression by sponging miRNA-503-5p, thereby maintaining the expression of PDCD4.

### Multiple miRNAs

The treatment SW480 CRC cells with Resveratrol (trans- 3,40,5-trihydroxystilbene), a poly phenolic, non-flavonoid antioxidant with reported tumour repressor effects, resulted in changes in the expression of numerous microRNAs, with 22 miRNAs being increased and 26 miRNAs decreased (263). Resveratrol treatment also resulted in a concomitant 60% increase of in the expression of the tumour suppressors PDCD4 and Phosphatase and tensin homolog (PTEN). The microRNAs predicted to target PDCD4 were miRNA16-1/17/21/23a/23b/160/181a2/424, which were decreased and miRNAs 340/497 which were increased. Further characterisation of how the expression of each of these miRNAs affect SW480 proliferation, migration and invasion, as well as PDCD4 expression were not forthcoming.

### miRNA-1260b

The expression of miRNA-1260b in CRC tumours showed the microRNA to be upregulated compared to normal tissue (264). The effects of this microRNA on cell fate were shown by transfection of the miRNA-1260b inhibitor into HCT-116 and SW480 cells which decreased proliferation and increased apoptosis. PDCD4 was identified as a predicted target of miRNA-1260b and a direct interaction was confirmed using luciferase assays. Examining the potential effect of miRNA-1260b expression on 5-fluoruracil (5-FU) resistance, it was found that independently, both 5-FU administration and transfection of miRNA-1260b inhibitor reduced proliferation and increased apoptosis, effects amplified in cells subjected to both treatments. However, these treatments also reduced the expression of PDCD4 protein, which is a counterintuitive finding as increased PDCD4 expression has extensively been associated with decreased proliferation and increased apoptosis. An explanation of this finding was not given.

## Future Direction

Although PDCD4 is a confirmed tumour suppressor, little is known about how the protein suppresses tumorigenesis. Primarily, there is a paucity of information on the identification of the targets of PDCD4 which modulate cell proliferation, differentiation and apoptosis. The advent of new generation sequencing has made it possible to identify most, if not all, of the mRNA transcriptome that is being expressed at a specific time and could be used to examine differences in gene expression between tumour cells where PDCD4 is suppressed and non-tumour cells expressing the tumour suppressor. Although this gives an indication of protein expression, not all mRNA is translated and therefore protein studies, including proteomic, are required to verify any observed changes. In addition, future research should examine how PDCD4 is discriminatory in inhibiting translation of specific proteins in the cytoplasm and gene expression in the nucleus. Mechanistically, it is known that PDCD4 binds to the translation initiation complex thereby inhibiting translation, but the expression of PDCD4 does not totally inhibit protein production as cells expressing the protein still function normally. Given that PDCD4 can be translocated to and from the nucleus, it could be surmised that translation is prevented from being inhibited by sequestering PDCD4 to the nucleus. However, the protein has been seen to be in both the cytoplasm and nucleus of normal cells. How PDCD4 does not indiscriminately inhibit translation requires investigation. Furthermore, it has been found that in many transformed cells, although downregulated, PDCD4 is cytoplasmic yet predominantly in the nucleus of equivalent quiescent controls, indicating that the protein acts as a tumour suppressor in the nucleus. How the protein modulates transcription is also unknown and should be examined.

Inflammation is positively linked with tumorigenesis in many cancers, especially those of the GI tract. Although PDCD4 is a tumour suppressor, it is associated with a pro-inflammatory state, whereas the oncomir miRNA-21, an inhibitor of PDCD4 expression, is thought to be anti-inflammatory. The relationships between PDCD4, miRNA-21, inflammation and tumorigenesis therefore need to be reconciled, which includes how miRNA-21 and possibly other microRNAs secreted in exosomes by activated macrophages in the tumour microenvironment during inflammation can downregulate the expression of epithelial PDCD4 and promote tumorigenesis.

Overall, although there is ever-increasing evidence that PDCD4 dysregulation may be integral to cellular transformation during tumorigenesis, relatively little is known about how the protein functions as a tumour suppressor and the regulation of its expression in cancers of the GI tract. Understanding these is fundamental in developing new therapies that utilise the tumour suppressor and its interacting proteins for treatment of patients with cancer.

## Conclusions

PDCD4 is a known tumour suppressor which can inhibit translation in the cytoplasm and affect gene transcription in the nucleus, potentially thereby modulating proliferation, differentiation and apoptosis. Although the exact mechanism of this is still elusive, numerous investigations have found decreased PDCD4 expression in many forms of transformed cells, suggesting an implicit role of the protein in the regulation of oncogenesis. As GI tract cancers have a high burden of disease, accounting for nearly a quarter of cancer-related deaths in 2020, information on the aetiology, propagation and dissemination of these cancers and the expression and explicit function of tumour suppressors such as PDCD4 in normal GI cells is invaluable. Although examining the downregulation of PDCD4 in tumours has become increasingly common, only a relatively few publications have shown how the expression of the protein is regulated. These include investigations examining post-translational modifications such as phosphorylation and ubiquitination and as reviewed here, downregulation of protein production by the inhibition and in some cases, degradation of PDCD4 mRNA by microRNAs. Generally, these investigations focus on specific microRNAs, identified in tumours using arrays or new generation sequencing as having the highest or near-highest expression compared to other miRNAs. With the exception of miRNA-21 which seems to be universally upregulated in GI tract cancers, the microRNA expression profiles from different investigations are often dissimilar. This may be accounted for by differences between the tumour samples, including cell type, stage, location and the tumour microenvironment. Examining the interactions of a single miRNA with PDCD4 is the first step in understanding how the tumour suppressor is potentially regulated *in vivo*, but analysis of the 3’-UTR of PDCD4 shows predicted binding of at least 80 different microRNAs, suggesting that regulation could be extremely complex. The expression of multiple miRNAs within tumours acting in concert is further complicated by the importation of exogenous miRNAs in exosomes secreted by non-tumour cells in the tumour microenvironment. Understanding how these and endogenous miRNAs are orchestrated will be fundamental in determining how tumour suppressors such as PDCD4 are regulated *in vivo*.

## Author Contributions

The author confirms being the sole contributor of this work and has approved it for publication.

## Conflict of Interest

The author declares that the research was conducted in the absence of any commercial or financial relationships that could be construed as a potential conflict of interest.

## Publisher’s Note

All claims expressed in this article are solely those of the authors and do not necessarily represent those of their affiliated organizations, or those of the publisher, the editors and the reviewers. Any product that may be evaluated in this article, or claim that may be made by its manufacturer, is not guaranteed or endorsed by the publisher.
